# Genotype and Phenotype Analysis in X-Linked Hypophosphatemia

**DOI:** 10.3389/fped.2021.699767

**Published:** 2021-08-09

**Authors:** Peong Gang Park, Seon Hee Lim, HyunKyung Lee, Yo Han Ahn, Hae Il Cheong, Hee Gyung Kang

**Affiliations:** ^1^Ministry of Health and Welfare, Sejong, South Korea; ^2^Department of Pediatrics, Uijeongbu Eulji Medical Center, Uijeongbu, South Korea; ^3^Department of Pediatrics, Kangwon National University Hopsital, Chuncheon, South Korea; ^4^Department of Pediatrics, Seoul National University College of Medicine, Seoul, South Korea; ^5^Department of Pediatrics, Seoul National University Children's Hospital, Seoul, South Korea; ^6^Kidney Research Institute, Seoul National University Medical Research Center, Seoul, South Korea; ^7^Department of Pediatrics, Hallym University Sacred Heart Hospital, Seoul, South Korea; ^8^Wide River Institute of Immunology, Seoul National University, Hongcheon, South Korea

**Keywords:** X-linked hypophosphatemic rickets, genotype, truncating mutation, Long-term follow up, genotype-phenotype analysis

## Abstract

**Background:** X-linked hypophosphatemia (XLH) is the most frequent form of hypophosphatemic rickets and is caused by mutations in the *PHEX* gene. We analyzed genotype-phenotype correlations in XLH patients with proven *PHEX* mutations.

**Methods:***PHEX* mutations were detected in 55 out of 81 patients who clinically presented with hypophosphatemic rickets. The patients were grouped into nontruncating (*n* = 9) and truncating (*n* = 46) mutation groups; their initial presentation as well as long-term clinical findings were evaluated according to these groups.

**Results:** Initial findings, including presenting symptoms, onset age, height standard deviation scores (SDS), and laboratory tests, including serum phosphate level and tubular resorption of phosphate, were not significantly different between the two groups (onset age: nontruncating mutation group, 2.0 years, truncating mutation group, 2.2 years; height SDS: nontruncating mutation group, −1.9, truncating mutation group, −1.7; serum phosphate: nontruncating mutation group, 2.5 mg/dL, truncating mutation group, 2.6 mg/dL). However, at their last follow-up, the serum phosphate level was significantly lower in patients with truncating mutations (nontruncating mutation group: 3.2 mg/dl, truncating mutation group: 2.3 mg/dl; *P* = 0.006). Additionally, 62.5% of patients with truncating mutations developed nephrocalcinosis at their last follow-up, while none of the patients with nontruncating mutations developed nephrocalcinosis (*P* = 0.015). Orthopedic surgery due to bony deformations was performed significantly more often in patients with truncating mutations (52.3 vs. 10.0%, *P* = 0.019).

**Conclusion:** Although considerable inconsistency exists regarding the correlation of truncating mutations and their disease phenotype in several other studies, we cautiously suggest that there would be genotype-phenotype correlation in some aspects of disease manifestation after long-term follow-up. This information can be used when consulting patients with confirmed XLH regarding their disease prognosis.

## Introduction

X-linked hypophosphatemia (XLH, Online Mendelian Inheritance in Man # 307800) is the most frequent form of hypophosphatemic rickets, with an estimated prevalence of one in 20,000 individuals in the general population (1). XLH is clinically characterized by renal hypophosphatemia, growth failure, skeletal deformity and dental abscesses (2, 3). XLH is caused by pathologic loss-of-function mutations in the *PHEX* (phosphate-regulating gene with homologies to endopeptidases on the X chromosome) gene, located on Xp22.1, which causes an elevation of circulating levels of fibroblast growth factor 23 (FGF23), which regulates renal phosphate reabsorption and the production of calcitriol (4). Increased levels of FGF23 suppress the activities of Na-phosphate cotransporter in both the kidney and small intestine and suppress the expression of 25(OH)D3 1-hydroxylase in the kidney (5, 6). The mechanism by which *PHEX* mutations lead to elevated levels of FGF23 remains unclear, although it is known that both are products of osteocytes (7). To date, various mutations in the *PHEX* gene, which consists of 22 short exons with very large intronic regions, have been reported. More than 400 types of *PHEX* variants have been annotated in the Human Gene Mutation Database (HGMD; http://www.hgmd.cf.ac.uk). These mutations, including frameshift mutations, missense mutations, intronic splice-site mutations, nonsense mutations, and deletions, are spread throughout the gene, with no identified specific hot spot.

The clinical history of XLH is heterogeneous, as the severity of growth failure and skeletal deformity varies between affected individuals. Additionally, a wide variety of dental abscesses and nephrocalcinoses have been reported among patients (2). Genotype-phenotype correlations are relatively well-established in several X-linked genetic diseases, such as Alport syndrome and Rett syndrome, and many of them have revealed that truncating mutations have a more severe phenotype than nontruncating mutations. Several studies have also reported a genotype-phenotype correlation in patients with XLH from Western countries (8–10). However, few data are available regarding the incidence and clinical manifestations of XLH in Asian populations, although a recent Chinese report analyzed some of them and revealed no genotype-phenotype correlation (11). Our institution also had previously reported hypophosphatemic rickets associated with eight types of *PHEX* gene mutations but found no phenotype-genotype correlation due to the small patient number (12); another Korean study at another institution analyzed several hereditary rickets and revealed that mutations in the C-terminal half of the *PHEX* gene were associated with a severe bony phenotype; however, this study did not focus on XLH (13).

Herein, we performed genotype-phenotype analysis in 57 Korean patients from 50 families with XLH with long-term follow-up data and compared the results with those of previous reports.

## Patients and Methods

### Study Participants and Their Clinical Information

In total, 55 patients from 48 unrelated families were enrolled in Seoul National University Children's Hospital. Genetic screening of the *PHEX* gene was performed in children who were clinically suspected to have hypophosphatemic rickets, who had persistent hypophosphatemia with normal/low PTH and low TRPi values; *PHEX* gene mutations were detected in 55 out of 81 patients screened. Medical records, including clinical, laboratory, and radiological data, of the patients were reviewed retrospectively. Eight cases have been published in previous reports of our institution (12).

### Genetic Study and Genotype-Phenotype Correlation

Genomic DNA was extracted from nucleated cells in peripheral blood and analyzed by Sanger sequencing. For the detection of punctual mutations, the 22 exons and intron-exon boundaries that comprise the *PHEX* gene were independently amplified by PCR using primers already described (12). To evaluate the genotype-phenotype correlation, the patients were grouped into nontruncating (*n* = 9) and truncating (*n* = 46) mutation groups according to their genotypes.

### Statistical Analyses

Qualitative data are described as frequencies, and quantitative data are described as medians and interquartile ranges (IQRs). Groups were compared with a *t*-test, chi-squared test, or Mann–Whitney *U*-test, as appropriate. A *P* < 0.05 was considered statistically significant. All statistical analyses were performed by using R-project version 4.0.2 (R core team, Vienna, Austria).

### Ethics Statement

This study was approved by the Institutional Review Board at Seoul National University Hospital (IRB No. 0812-002-264 and 2007-177-114). Informed consent for genetic testing was obtained from the patients or parents, and the requirement for written informed consent was waived due to the anonymous and retrospective nature of the study.

## Results

### Genotypes

The genetic study revealed missense mutations in seven patients, in-frame insertions/deletions in two patients, nonsense mutations in 15 patients, frame-shifting insertions/deletions in 15 patients, large deletions in five patients, and abnormal splicing in 11 patients (Table 1). In all, nine patients—all index cases—had nontruncating mutations, including missense mutations and in-frame insertion/deletion mutations. Forty-six patients, of whom 39 were index cases, had truncating mutations, including nonsense mutations, frame-shifting insertions/deletions and abnormal splicing mutations. Detailed genotypic information is shown in Supplementary Table 1. Of the 48 mutations found in our study, 20 of the variants were listed as pathogenic or likely pathogenic in the ClinVar database (https://www.ncbi.nlm.nih.gov/clinvar); three of them were previously reported mutations. *In silico* test using MutationTaster application (http://mutationtaster.org) predicted all of the novel non-truncating variants to be disease-causing mutations.

**Table 1:** Genotype of patients with XLH.

**Mutations**	**Index cases**	**Family members**	**Total**
Missense	7	0	7
Nonsense	12	3	15
Abnormal splicing	10	1	11
Frame-shifting insertion/deletion	13	2	15
In-frame ins/del	2	0	2
Large deletion	4	1	5
Total	48	7	55

### Clinical Findings According to Their Genotypes

The clinical features of 48 index patients are shown in Table 2. The median age at onset was 2.1 years (IQR; 1.4–3.2). A total of 64.3% of them were found to have initial symptoms of bow legs. Their initial serum phosphate level was 2.5 (IQR; 2.2–2.9) mg/dL, and renal tubular reabsorption of phosphate (TRPi) was 69.0 (IQR; 56.0–82.0).

**Table 2:** Initial presentation of index patients with XLH according to genotype.

	**Total (*n* = 48)**	**Nontruncating mutation (*n* = 9)**	**Truncating mutation (*n* = 39)**	***P*-value**
Onset age (years)	2.1 (1.4 to 3.2)	2.0 (1.2 to 2.6)	2.2 (1.6 to 3.2)	0.561
Male sex	19 (39.6%)	4 (44.4%)	15 (38.5%)	>0.999
Height SDS	−1.8 (−3.0 to −0.8)^*^^n = 44^	−1.9 (−2.2 to −1.3)	−1.7 (−3.1 to −0.8)	0.793
Serum Pi (mg/dL)	2.5 (2.2 to 2.9)	2.5 (2.4 to 2.9)	2.6 (2.2 to 2.9)	0.672
Serum Ca (mg/dL)	9.4 (9.1 to 9.7)	9.4 (9.1 to 9.6)	9.4 (9.2 to 9.8)	0.750
Serum ALP (IU/L)	598.5 (434.5 to 801.0)	588.0 (495.0 to 727.0)	609.0 (434.5 to 801.0)	0.916
Serum 25(OH)D_3_ (ng/mL)	29.0 (20.1 to 54.5)^*^^n = 31^	22.7 (19.5 to 24.4)	36.0 (22.6 to 60.5)	0.023
Serum PTH (pg/mL)	50.5 (32.6 to 85.5)^*^^n = 36^	61.7 (50.0 to 83.0)	45.5 (31.0 to 88.0)	0.235
TRPi (%)	69.0 (56.0 to 82.0)^*^^n = 37^	77.0 (61.0 to 86.6)	67.0 (56.0 to 79.0)	0.362
TmP/GFR (mg/dL)	2.2 (1.8 to 2.6)^*^^n = 37^	2.4 (1.9 to 2.7)	2.1 (1.8 to 2.5)	0.362
Urine Ca/Cr (mg/mg)	0.05 (0.02 to 0.10)^*^^n = 37^	0.04 (0.02 to 0.08)	0.05 (0.03 to 0.13)	0.644

* *Number of patients with available data were presented*.

*SDS, standard deviation score; ALP, alkaline phosphatase; PTH, parathyroid hormone; TRPi, tubular resorption of phosphate; TmP/GFR, tubular maximum reabsorption of phosphate Cr, creatinine*.

Among 9 index patients with nontruncating mutations, 8 (88.9%) patients presented with bow legs at the median age of 2.3 years (IQR; 1.3–2.9 years), and one presented with incidental laboratory abnormalities. Among 39 index patients with truncating mutations, 21 (53.8%) patients presented with bow legs at the median age of 2.0 years (IQR; 1.5–3.0 years), and two presented with incidental laboratory abnormalities. The median age at onset, initial serum phosphate level, TRPi, and initial height standard deviation score (SDS) were not significantly different between the two groups (Table 2). When we confined our analysis to 22 male patients, there was not significantly different initial clinical findings between truncating mutation and nontruncating mutation patients (Supplementary Table 2).

### Long-Term Follow-Up

Enrolled patients were followed up at a median of 13.1 (IQR; 5.1–22.5) years, and at their last follow-up, the patients' median age was 16.1 (IQR; 8.3–27.9) (Table 3). Serum Pi was significantly lower in patients with truncating mutations; however, their last height SDS was not significantly different between the two groups. Twenty-three (44.2%) patients underwent orthopedic surgery, and 25 (54.3%) had nephrocalcinosis. Interestingly, none of the nine patients with nontruncating mutations had nephrocalcinosis at their last follow-up, whereas 25 (62.5%) patients with truncating mutations had nephrocalcinosis at their last follow-up (*P* = 0.015). Additionally, significantly more patients with truncating mutations underwent orthopedic surgery due to bone deformity than patients with nontruncating mutations (0 vs. 52.3%, *P* = 0.019). When we confined our analysis to male patients, lower serum phosphorus was also seen in patients with truncating mutation (Supplementary Table 3).

**Table 3:** Long term follow-up of patients with XLH according to genotype.

	**Total (*n* = 55)**	**Nontruncating mutation (*n* = 9)**	**Truncating mutation (*n* = 46)**	***P-*value**
Age (years)	16.1 (8.3 to 27.9)	12.1 (7.0 to 15.5)	17.8 (8.6 to 29.5)	0.191
Serum Pi (mg/dL)	2.3 (1.9 to 2.9)^*^^n = 54^	3.2 (2.4 to 3.3)	2.3 (1.9 to 2.6)	0.006
Serum Ca (mg/dL)	9.3 (9.1 to 9.8)^*^^n = 54^	9.5 (9.2 to 9.6)	9.3 (9.1 to 9.8)	0.383
ALP (IU/L)	209.5 (115.0 to 472.0)^*^^n = 54^	417.0 (234.0 to 495.0)	174.0 (115.0 to 441.0)	0.214
Height SDS	−2.0 (−2.8 to −1.2)^*^^n = 43^	−1.7 (-2.4 to −1.2)	−2.2 (−2.9 to −1.2)	0.522
Nephrocalcinosis	25 (54.3%)^*^^n = 46^	0 (0.0%)	25 (62.5%)	0.015
Orthopedic surgery	23 (44.2%)^*^^n = 52^	0 (0.0%)	23 (52.3%)	0.019

* *Number of patients with available data were presented*.

*SDS, standard deviation score; ALP, alkaline phosphatase*.

## Discussion

In our study, we found that patients with truncating mutations had significantly lower serum Pi and a high proportion of nephrocalcinosis during their long-term follow-up period, although clinical manifestations were not significantly different at their initial presentation. We compared our results with those of other previously reported studies that provided patient clinical characteristics, including height and laboratory findings, along with their genotype (Table 4). Compared with three previous studies, the onset age and initial laboratory results of our study were not significantly different. Out of three previously reported genotype-phenotype studies, none of them reported significant differences in initial clinical characteristics between types of mutations, although one study reported that patients with truncating mutations had lower TRPis than patients without truncating mutations (9). In addition, one study reported long-term follow-up results and found no significant difference between the two groups; however, the study included only 21 patients (10). One study conducted a functional study with 10 known mutations (three truncating mutations; seven nontruncating mutations) and revealed no clear difference in protein expression and endopeptidase activity between mutation types. Another study concentrated on skeletal manifestations and found no correlation between disease severity and the type or location of *PHEX* mutations (8). We previously reported negative genotype-phenotype correlation results with eight patients, which were included in this study; a follow-up study with more recruited patients in this study found a new correlation at their long-term follow-up. Further study with a large number of XLH patients is warranted to clearly determine this association; however, on the basis of our results from the largest retrospective study where correlation was analyzed to date, we carefully suggest that truncating mutations are associated with a severe phenotype. Of course, there was also phenotypical variability within patients with truncating mutation. It is known that nonsense mutation is recognized by nonsense-mediated decay (NMD) system and degraded; mutations that escape NMD could have a better function and these may influence their variance of the phenotype (14).

**Table 4:** Comparison of association in *PHEX* mutation genotype and phenotype in several studies.

**Study (reference)**	**At diagnosis**										**Last follow-up**							
	**Truncating mutation**					**Nontruncating mutation**					**Truncating mutation**				**Nontruncating mutation**			
	**No**.	**Age**	**Ht SDS**	**Pi**	**TRPi**	**No**.	**Age**	**Ht SDS**	**Pi**	**TRPi**	**Age**	**Ht SDS**	**Pi**	**NC**	**Age**	**Ht SDS**	**Pi**	**NC**
This study	46	2.2	−1.7	2.6	67	9	2.0	−1.9	2.5	77	17.8	−2.2	2.3^*^	63%^*^	12.1	−1.7	3.2^*^	0%^*^
Morey et al. (9)	30	3.43	−2.81	2.61	61.4^*^	6	1.82	−1.48	2.81	80.1^*^	–	–	–	16%	–	–	–	0%–
Rafaelsen et al. (10)	9	0.92	−1.68	2.79	–	12	2.0	0.87	2.48	–	7.17	−1.39	–	54.50%	12.54	−2	–	55.60%
Zheng et al. (11)	39	2.2	−2.2	2.4	69	14	2.3	−2.1	2.4	72	–	–	–	–	–	–	–	–

*Ht SDS, standard deviation score of height; Pi, serum phosphate; TRPi, tubular resorption of phosphate; NC, nephrocalcinosis*.

*Units: Age, year; Pi, mg/dL; TRPi, %; NC, %*.

* *Significant difference between two groups of patients with available data were presented*.

In particular, we found a significant difference in the development of nephrocalcinosis between the two groups; none of the patients with nontruncating mutations had nephrocalcinosis, whereas over half of the patients with truncating mutations developed nephrocalcinosis. Another study also reported that none of the patients with nontruncating mutations developed nephrocalcinosis, although the association was not statistically significant due to the small sample size (9). Another study reported a similar incidence of nephrocalcinosis between the two groups; however, its sample size was small, and the follow-up period of patients was longer in patients with nontruncating mutations (10). As it is known that hypercalciuria, hyperoxaluria, and hyperphosphaturia are important risk factors for the progression of nephrocalcinosis (15), the degree of phosphaturia, which was significantly different between the two groups in one previous study, can be an important risk factor. Although we could not take the severity of nephrocalcinosis into account due to the retrospective nature of our study and the progression of nephrocalcinosis can be confounded by other factors such as urine oxalate or treatment with the phosphate and active vitamin D, our study suggests that patients with truncating mutations should undergo more aggressive surveillance of nephrocalcinoses with kidney ultrasonography at an early age.

This study has inherent limitations: it is a retrospective medical review and cannot represent the overall XLH population, since it is a single-center study. Additionally, the timing of treatment varies among patients and can affect their long-term follow-up results. Due to the lack of a sufficient follow-up period, dental prognosis, such as abscess formation, was not fully elucidated. Furthermore, we could not conduct analysis regarding age-specific standard values of serum phosphate, TRPi, and ALP. However, we supposed that our findings that serum phosphate level was significantly different while their age was not significantly different were meaningful. Also, due to the nature of the retrospective study, we could not further investigate whether the skewed inactivation is observed in female patients, such as using mRNA microarray or whole-exome sequencing technique, which can provide further insight into X-linked disease condition. Finally, as we did not conduct mixed ligation-dependent probe amplification of *PHEX*, we might have missed some patients with large deletion. Nevertheless, our study has the strength of being, to our knowledge, the largest study that conducted genotype-phenotype analysis in patients with *PHEX* mutations, although the number of patients with nontruncated mutation was relatively small and the overall sample size was not large enough to ensure statistical power. Further prospective studies with longer follow-up periods should be conducted to demonstrate a more comprehensive prognosis in XLH patients. International collaboration of clinical and genetic data of XLH patients using the *PHEX* repository can accomplish this purpose.

In conclusion, we presented a genotype-phenotype correlation study with a relatively large population in a Korean population, and we compared our results with those other studies regarding the genotype-phenotype correlation in patients with XLH. Although considerable nonconsistency exists regarding the correlation of truncating mutations and their disease phenotype, we cautiously suggest that there would be a positive genotype-phenotype correlation in some aspects of disease manifestation. Although an antibody to neutralize FGF23, namely, burosumab (16), has recently been approved in an increasing number of countries, multiple daily administrations of oral phosphate to offset renal loss with active vitamin D supplementation are the mainstay of therapy so far worldwide. If we can predict disease severity and the risk of progression, we can establish adequate treatment and follow-up plans for these patients according to their genotype at the time of their diagnosis. This information can also be used when consulting patients with confirmed XLH regarding their disease prognosis.

## Data Availability Statement

The original contributions presented in the study are included in the article/Supplementary Materials, further inquiries can be directed to the corresponding author/s.

## Ethics Statement

The studies involving human participants were reviewed and approved by Institutional Review Board at Seoul National University Hospital (IRB No. 0812-002-264 and 2007-177-114). Written informed consent from the participants' legal guardian/next of kin was not required to participate in this study in accordance with the national legislation and the institutional requirements.

## Author Contributions

All authors listed have made a substantial, direct and intellectual contribution to the work and approved it for publication.

## Conflict of Interest

The authors declare that the research was conducted in the absence of any commercial or financial relationships that could be construed as a potential conflict of interest.

## Publisher's Note

All claims expressed in this article are solely those of the authors and do not necessarily represent those of their affiliated organizations, or those of the publisher, the editors and the reviewers. Any product that may be evaluated in this article, or claim that may be made by its manufacturer, is not guaranteed or endorsed by the publisher.

## References

[1] Tenenhouse HS. X-linked hypophosphataemia: a homologous disorder in humans and mice. Nephrol Dial Transplant. (1999) 14:333–41. 10.1093/ndt/14.2.333 10069185

[2] Carpenter TO Imel EA Holm IA Jan de Beur SM Insogna KL. A clinician's guide to X-linked hypophosphatemia. J Bone Miner Res. (2011) 26:1381–8. 10.1002/jbmr.340 21538511 PMC3157040

[3] Skrinar A Dvorak-Ewell M Evins A Macica C Linglart A Imel EA. The lifelong impact of X-linked hypophosphatemia: results from a burden of disease survey. J Endocr Soc. (2019) 3:1321–34. 10.1210/js.2018-00365 31259293 PMC6595532

[4] Francis F Hennig S Korn B Reinhardt R de Jong P Poustka A. A gene (PEX) with homologies to endopeptidases is mutated in patients with X-linked hypophosphatemic rickets. The HYP Consortium. Nat Genet. (1995) 11:130–6. 10.1038/ng1095-130 7550339

[5] Saito H Kusano K Kinosaki M Ito H Hirata M Segawa H. Human fibroblast growth factor-23 mutants suppress Na+-dependent phosphate co-transport activity and 1α,25-dihydroxyvitamin D3 production. J Biol Chem. (2003) 278:2206–11. 10.1074/jbc.M207872200 12419819

[6] Shimada T Mizutani S Muto T Yoneya T Hino R Takeda S. Cloning and characterization of FGF23 as a causative factor of tumor-induced osteomalacia. Proc Natl Acad Sci U S A. (2001) 98:6500–5. 10.1073/pnas.101545198 11344269 PMC33497

[7] Ruchon AF Marcinkiewicz M Siegfried G Tenenhouse HS DesGroseillers L Crine P. Pex mRNA is localized in developing mouse osteoblasts and odontoblasts. J Histochem Cytochem. (1998) 46:459–68. 10.1177/002215549804600405 9524191

[8] Holm IA Nelson AE Robinson BG Mason RS Marsh DJ Cowell CT. Mutational analysis and genotype-phenotype correlation of the PHEX gene in X-linked hypophosphatemic rickets. J Clin Endocrinol Metab. (2001) 86:3889–99. 10.1210/jcem.86.8.7761 11502829

[9] Morey M Castro-Feijoo L Barreiro J Cabanas P Pombo M Gil M. Genetic diagnosis of X-linked dominant Hypophosphatemic Rickets in a cohort study: tubular reabsorption of phosphate and 1,25(OH)2D serum levels are associated with PHEX mutation type. BMC Med Genet. (2011) 12:116. 10.1186/1471-2350-12-116 21902834 PMC3189111

[10] Rafaelsen S Johansson S Raeder H Bjerknes R. Hereditary hypophosphatemia in Norway: a retrospective population-based study of genotypes, phenotypes, and treatment complications. Eur J Endocrinol. (2016) 174:125–36. 10.1530/EJE-15-0515 26543054 PMC4674593

[11] Zheng B Wang C Chen Q Che R Sha Y Zhao F. Functional characterization of PHEX gene variants in children with X-linked hypophosphatemic rickets shows no evidence of genotype-phenotype correlation. J Bone Miner Res. (2020) 35:1718–25. 10.1002/jbmr.4035 32329911

[12] Cho HY Lee BH Kang JH Ha IS Cheong HI Choi Y. A clinical and molecular genetic study of hypophosphatemic rickets in children. Pediatr Res. (2005) 58:329–33. 10.1203/01.PDR.0000169983.40758.7B 16055933

[13] Song HR Park JW Cho DY Yang JH Yoon HR Jung SC. PHEX gene mutations and genotype-phenotype analysis of Korean patients with hypophosphatemic rickets. J Korean Med Sci. (2007) 22:981–6. 10.3346/jkms.2007.22.6.981 18162710 PMC2694264

[14] Khajavi M Inoue K Lupski JR. Nonsense-mediated mRNA decay modulates clinical outcome of genetic disease. Eur J Hum Genet. (2006) 14:1074–81. 10.1038/sj.ejhg.5201649 16757948

[15] Baroncelli GI Bertelloni S Sodini F Galli L Vanacore T Fiore L. Genetic advances, biochemical and clinical features and critical approach to treatment of patients with X-linked hypophosphatemic rickets. Pediatr Endocrinol Rev. (2004) 1:361–79. 16437029

[16] Insogna KL Briot K Imel EA Kamenicky P Ruppe MD Portale AA. A randomized, double-blind, placebo-controlled, phase 3 trial evaluating the efficacy of burosumab, an anti-FGF23 antibody, in adults with X-linked hypophosphatemia: week 24 primary analysis. J Bone Miner Res. (2018) 33:1383–93. 10.1002/jbmr.3475 29947083

