# Supplementary material for: Genotype and Phenotype Analysis in X-Linked Hypophosphatemia
Source: Front Pediatr. 2021 Aug 9;9:699767. doi: 10.3389/fped.2021.699767 (PMC8382157; doi:10.3389/fped.2021.699767)
Supplement: Supplementary file 1 [file Data_Sheet_1.docx]

Supplementary Material

Supplementary Table 1. Detailed genotypic information of patients with *PHEX* mutation

| Case ID | Sex | cDNA mutation (RefSeq: NM_000444.6) | | | Protein | | Exon | Novelty | ClinVar or reference | *In silico* prediction^3^ |
| --- | --- | --- | --- | --- | --- | --- | --- | --- | --- | --- |
| ***Missense mutation*** | | |  |  | |  |  |  |  |  |
| 1 | M | c.1037A>G | | | p.Tyr(TAC)346Cys(TGC) | | 9 |  | Likely pathogenic |  |
| 2 | F | c.1602C>T | | | p.Pro(CCG)534Leu(CTG) | | 15 |  | Rowe, 1997 (1) |  |
| 3 | M | c.1638C>G | | | p.Asn(AAC)546Lys(AAG) | | 15 | Yes |  | Deleterious |
| 4 | M | c.1715G>A | | | p.Gly(GGT)572Asp(GAT) | | 17 |  | Pathogenic |  |
| 5 | F | c.1717G>C | | | p.Ala(GCT)573Pro(CCT) | | 17 |  | Pathogenic |  |
| 6 | F | c.2078G>T | | | p.Cys(TGC)693Phe(TTC) | | 21 |  | Durmaz, 2015 (2) |  |
| 7 | F | c.2149G>T | | | p.Val(GTC)717Phe(TTC) | | 22 |  | VUS | Deleterious |
| ***Nonsense mutation*** | | |  |  | |  |  |  |  |  |
| 8 | M | c.58C>T | | | p.Arg(CGA)20*(TGA) | | 1 |  | Pathogenic |  |
| 9 | F | c.58C>T | | | p.Arg(CGA)20*(TGA) | | 1 |  | Pathogenic |  |
| 10 | M | c.322A>T | | | p.Arg(AGA)108*(TGA) | | 3 | Yes |  | Deleterious |
| 11 | F | c.871C>T | | | p.Arg(CGA)291*(TGA) | | 8 |  | Pathogenic |  |
| 11-1^1^ | F |  | | |  | |  |  |  |  |
| 12 | M | c.931C>T | | | p.Gln(CAG)311*(TAG) | | 8 |  | Rowe, 1997 (1) |  |
| 13 | F | c.931C>T | | | p.Gln(CAG)311*(TAG) | | 8 |  | Rowe, 2015 (1) |  |
| 14 | M | c.1363G>T | | | p.Glu(GAG)455*(TAG) | | 12 |  | Pathogenic |  |
| 14-1^1^ | F |  | | |  | |  |  |  |  |
| 15 | F | c.1536T>A | | | p.Tyr(TAT)512*(TAA) | | 14 | Yes |  | Deleterious |
| 15-1^2^ | M |  | | |  | |  |  |  |  |
| 16 | F | c.1645C>T | | | p.Arg(CGA)549*(TGA) | | 15 |  | Pathogenic |  |
| 17 | F | c.1971C>G | | | p.Tyr(TAC)657*(TAG) | | 20 |  | Pathogenic |  |
| 18 | F | c.2104C>T | | | p.Arg(CGA)702*(TGA) | | 21 |  | Pathogenic |  |
| 19 | F | c.2239C>T | | | p.Arg(CGA)747*(TGA) | | 22 |  | Pathogenic |  |
| ***Abnormal splicing*** | | |  |  | |  |  |  |  |  |
| 20 | F | c.436+5G>A | | | IVS4+5 G>A | |  |  | VUS | Deleterious |
| 21 | F | c.850-2A>G | | | IVS7-2 A>G | |  |  | Conflicting | Deleterious |
| 22 | F | c.1173+2T>A | | | IVS10+2 T>A | |  | Yes |  | Deleterious |
| 23 | M | c.1586+2T>G | | | IVS14+2 T>G | |  |  | Pathogenic |  |
| 24 | F | c.1646-1 G>A | | | IVS15-1 G>A | |  | Yes |  | Deleterious |
| 25 | F | c.1645+1G>A | | | IVS15+1 G>A | |  |  | Pathogenic |  |
| 26 | M | c.1701-1delG | | | IVS16-1delG | |  |  | Likely pathogenic |  |
| 27 | F | c.1700+1G>A | | | IVS16+1 G>A | |  |  | Pathogenic |  |
| 28 | F | c.2148-1G>A | | | IVS21-1 G>A | |  | Yes |  | Deleterious |
| 29 | M | c.2071-2A>G | | | IVS20-2 A>G | |  |  | Pathogenic |  |
| 29-1^2^ | M |  | | |  | |  |  |  |  |
| ***Frame-shifting insertion/deletion*** | | | |  | |  |  |  |  |  |
| 30 | F | c.15_16delAG | | | p.Gly(GGG)6Glu(GAG) | | 1 |  | Pathogenic |  |
| 31 | F | c.130_145dupCTCTTAAGTCTCCAAG | | | p.G43 | | 2 | Yes |  |  |
| 32 | F | c.208_212delGTAAA | | | p.Val(GTA)70Ser(TCT) | | 3 |  | Pathogenic |  |
| 33 | F | c.208_212delGTAAA | | | p.Val(GTA)70Ser(TCT) | | 3 |  | Pathogenic |  |
| 34 | M | c.1082delC | | | p.Ile(ATT)362Leu(TTG) | | 10 | Yes |  |  |
| 34-1^2^ | F |  | | |  | |  |  |  |  |
| 35 | F | c.1177_1178delAT | | | p.Ile(ATC)393Pro(CCA) | | 11 | Yes |  |  |
| 35-1^1^ | F |  | | |  | |  |  |  |  |
| 36 | M | c.1177_1178delAT | | | p.Ile(ATC)393Pro(CCA) | | 11 | Yes |  |  |
| 37 | F | c. 1331delG | | | p.Ala(GCC)445Pro(CCT) | | 12 | Yes |  |  |
| 38 | F | c.1526delC | | | p.Thr(ACT)509Ile(ATC) | | 14 | Yes |  |  |
| 39 | M | c.1585_1586delGA | | | p.Glu(GAG)529Val(GTG) | | 14 | Yes |  |  |
| 40 | M | c.1996_2008delCAGGGACTTGAGG | | |  | | 20 | Yes |  |  |
| 41 | F | c.2171_2172delTT | | | p.Phe(TTT)724StopTGA) | | 22 |  | Likely pathogenic |  |
| 42 | M | c.2226_2230dupCATGG | | | p.Met(ATG)743Leu(CTC) | | 22 | Yes |  |  |
| ***In-frame insertion/deletion*** | | | |  | |  |  |  |  |  |
| 43 | F | c.996_998delCAT | | | p.Ile(ATC)333del | | 9 | Yes |  | Deleterious |
| 44 | M | c.1952_1963delGGGAAGCTTTTA | | | p.E652-R655del4 | | 19 | Yes |  | Deleterious |
| ***Large deletion*** | | |  |  | |  |  |  |  |  |
| 45 | M | Deletion covering exon 1, 2, and 3 | | | | |  |  |  |  |
| 45-1^1^ | F |  | | | | |  |  |  |  |
| 46 | F | deletion of exons 8 and 9 | | |  | |  |  |  |  |
| 47 | M | deletion of exons 10 and 11 | | |  | |  |  |  |  |
| 48 | M | Deletion covering exon 13~22 | | | | |  |  |  |  |

* Abbreviation: cDNA, complementary DNA; IVS, intervening sequence; VUS, variance of unknown significance

^1^ denotes parent; ^2^ denotes sibling; ^3^In silico prediction was conducted using MutationTaster (http://mutationtaster.org)

Supplementary Table 2. Initial presentation of male index patients with XLH according to genotype

|  | **Total (n=19)** | **Nontruncating mutation (n=4)** | **Truncating mutation (n=15)** | **P value** |
| --- | --- | --- | --- | --- |
| **Onset age (years)** | 2.2 (1.7 -3.5) | 1.5 ( 0.8 - 2.6) | 2.4 ( 1.7 - 4.3) | 0.211 |
| **Height SDS** | -2.0 (-3.1 - -1.7) ^*n = 17^ | -1.4 (-2.1 - 0.2) | -2.1 (-3.2 - -1.9) | 0.212 |
| **Serum Pi (mg/dL)** | 2.8 (2.2 -3.0) | 2.8 ( 2.5 - 3.0) | 2.4 ( 2.1 - 2.9) | 0.339 |
| **Serum Ca (mg/dL)** | 9.4 (9.1 -9.7) | 9.3 ( 8.8 -10.1) | 9.4 ( 9.2 - 9.6) | 0.92 |
| **Serum ALP (IU/L)** | 588.0 (465.5 -905.5) | 657.5 (541.5 -864.5) | 586.0 (393.0 -905.5) | 0.53 |
| **Serum 25(OH)D_3_ (ng/mL)** | 27.0 (19.5 -35.5) ^*n = 11^ | 20.8 (18.9 -22.7) | 29.0 (20.1 -36.0) | 0.436 |
| **Serum PTH (pg/mL)** | 50.0 (32.6 -79.5) ^*n = 15^ | 56.4 (53.2 -92.2) | 38.5 (30.7 -79.5) | 0.295 |
| **TRPi (%)** | 67.0 (51.0 -80.5) ^*n = 16^ | 82.0 (57.0 -88.0) | 65.0 (56.0 -76.0) | 0.521 |
| **TmP/GFR (mg/dL)** | 2.1 (1.6 -2.5) | 2.6 ( 1.8 - 2.8) | 2.0 ( 1.8 - 2.4) | 0.521 |
| **Urine Ca/Cr (mg/mg)** | 0.05 (0.02 -0.09) ^*n = 17^ | 0.02 ( 0.02 - 0.06) | 0.06 ( 0.04 - 0.09) | 0.527 |

* Number of patients with available data were presented.

Abbreviation: SDS; standard deviation score; ALP; alkaline phosphatase; PTH; parathyroid hormone; TRPi; tubular resorption of phosphate; TmP/GFR, tubular maximum reabsorption of phosphate; Cr, creatinine

Supplementary Table 3. Long term follow-up of male patients with XLH according to genotype

|  | **Total (n=19)** | **Nontruncating mutation (n=4)** | **Truncating mutation (n=15)** | ***P* value** |
| --- | --- | --- | --- | --- |
| **Age (years)** | 12.7 (5.7 -26.3) | 13.8 ( 8.8 -19.3) | 12.7 ( 5.7 -29.9) | 0.885 |
| **Serum Pi (mg/dL)** | 2.7 (1.8 -3.2) | 3.2 ( 3.1 - 3.5) | 2.5 ( 1.8 - 2.8) | 0.014 |
| **Serum Ca (mg/dL)** | 9.3 (9.1 -9.8) | 10.1 ( 9.3 -10.7) | 9.2 ( 9.1 - 9.6) | 0.192 |
| **ALP (IU/L)** | 417.0 (166.0 -515.0) | 383.0 (222.0 -662.5) | 439.0 (166.0 -515.0) | >0.999 |
| **Height SDS** | -2.3 (-2.5 - -1.2) ^*n = 14^ | -1.4 (-1.8 - -0.6) | -2.5 (-2.8 - -2.2) | 0.055 |
| **Nephrocalcinosis** | 10 (58.8%)^*n = 17^ | 0 ( 0.0%) | 10 (71.4%) | 0.102 |
| **Orthopedic surgery** | 8 (42.1%) | 0 ( 0.0%) | 8 (53.3%) | 0.177 |

* Number of patients with available data were presented.

Abbreviation: SDS; standard deviation score; ALP; alkaline phosphatase

Reference

1. Rowe PS, Oudet CL, Francis F, Sinding C, Pannetier S, Econs MJ, et al. Distribution of mutations in the PEX gene in families with X-linked hypophosphataemic rickets (HYP). Hum Mol Genet. 1997;6(4):539-49.

2. Durmaz E, Zou M, Al-Rijjal RA, Baitei EY, Hammami S, Bircan I, et al. Novel and de novo PHEX mutations in patients with hypophosphatemic rickets. Bone. 2013;52(1):286-91.
